# Predicting and remembering the behaviors of social targets: how prediction accuracy affects episodic memory

**DOI:** 10.1186/s40359-022-00801-z

**Published:** 2022-04-09

**Authors:** Onyinye J. Udeogu, Andrea N. Frankenstein, Allison M. Sklenar, Pauline Urban Levy, Eric D. Leshikar

**Affiliations:** University of Illinois at Chicago, 1007 West Harrison Street (M/C 285), Chicago, IL 60607 USA

**Keywords:** Future thinking, Episodic memory, Predictions, Future simulations

## Abstract

**Background:**

Decades of research has investigated the relationship between memory and future thinking. Although some of this work has shown that memory forms the basis of making predictions about the future, less work has investigated how the outcome of those predictions (whether consistent or inconsistent with what one predicts) is later remembered. Limited past works suggests that memory for outcomes that are consistent with what one predicts are better remembered that predictions that are inconsistent. To advance understanding of the relationship between episodic memory and future thinking, the current investigation examines how the outcome of predictions affects memory after the predicted events takes place.

**Methods:**

In this experiment, participants first learned trait information about social targets. Then, participants imagined scenarios involving targets and the self (i.e., the participant) and made predictions about which behaviors targets would perform based on the trait information associated with targets participants learned earlier. Participants were then told the behaviors the targets actually performed (i.e., prediction outcome), which was either *consistent* or *inconsistent* with predictions, before then taking a memory test for prediction outcomes (what the social target actually did).

**Results:**

Results showed memory for prediction-consistent outcomes was better than for prediction-inconsistent outcomes, suggesting people exhibit enhanced memory for events that are in line with predictions based on existing contents of memory (e.g., what one knows; schemas), which is in line with the limited past work in this domain.

**Conclusion:**

Overall, finding better memory for prediction-consistent outcomes may reflect an adaptive function in memory, where people show enhanced memory for episodes when they play out as predicted, and aligned with the current contents of memory.

## Introduction

Future thinking has been the focus of increasing research over recent decades [1, 2]. Some of this work has identified a striking relationship between future thinking and memory [3–9], such as neuroimaging evidence that episodic memory and future thinking processes activate similar, overlapping areas in the brain [6, 7, 10], and other work demonstrating the contents of episodic memory (e.g., memory for past experiences) are used to generate new mental simulations or predictions of possible future events [11, 12]. Although future thinking research has explored how episodic memory is used in service of making predictions, there has been less work to understand how the outcomes of those predictions (after the predicted event occurs) are subsequently stored in memory. To understand the relationship between future thinking and episodic memory more clearly, the current investigation examines predictions and how the outcomes of those predictions (i.e., *prediction outcomes*) affect memory after the predicted event occurs. Specifically, we examine memory for events that occur as predicted (henceforth called *prediction-consistent outcomes*) as well as memory for events that do not occur as predicted (e.g., *prediction-inconsistent outcomes*). We do so in a task that involves participants making predictions about social targets, given that people frequently make predictions about the behaviors of others in social contexts [13].

To inform potential hypotheses on how prediction-consistent versus prediction-inconsistent outcomes might be represented in episodic memory after predicted events occur, it is first useful to survey past work in experiments that do not involving predictions on how consistency (whether new information is consistent or inconsistent with prior information) affects memory for social targets. This work has shown that memory for information that is consistent with prior information is sometimes better remembered that information that is inconsistent ([14–25]; though see below for work showing better memory for inconsistent information). For instance, in one investigation participants saw behaviors performed by members of two different fraternities. In the first part of the experiment, participants learned that members of the first fraternity were extraverted, whereas members of the second fraternity were introverted. Then, participants were shown additional behaviors of members of both fraternities that contained both extraverted and introverted behaviors. Results indicated that participants remembered more extraverted behaviors for members of the “extraverted” fraternity, and more introverted behaviors for members of the “introverted” fraternity, suggesting that participants were remembering details aligned with their prior knowledge [23]. Interestingly, past studies that have found better memory for consistent relative to inconsistent information about targets have often explained their findings through schema accounts, which suggest that people use the contents of their memory (e.g., schemas) to process new information about targets [26–28]. Specifically, schema accounts posit that when new information is aligned with memory stores (e.g., schemas), it is easier to integrate that information than inconsistent information, which leads to improved memory for consistent information. Turning to prediction outcomes, based on this past work, it is possible to hypothesize that memory for prediction-consistent outcomes would be better than prediction-inconsistent outcomes, in line with the idea that information consistent with schematic representations shows an advantage in memory. Given that schemas generally represent adequate understanding about the world, it may be adaptive to strongly rely on existing schematic representations when processing new information, such as new information about targets.

In one of the few studies that has directly examined the relationship between prediction outcome and episodic memory, Frankenstein et al. [29] investigated memory for prediction-consistent versus prediction-inconsistent outcomes. In that experiment, participants learned trait information about social targets (e.g., target is highly extraverted), and then made predictions about social targets’ future behaviors. After making predictions, participants were then shown the behavior the target actually performed (i.e., prediction outcome) which was either consistent or inconsistent with predictions. Data from that experiment showed enhanced memory for prediction-consistent relative to prediction-inconsistent outcomes, which fits with past work showing enhanced memory for consistent information [14–18, 20, 21, 23–25, 30], and further aligned with schema accounts of how information about targets is stored in memory [26–28]. Although the Frankenstein, et al. [29] findings showed a clear advantage in memory for prediction-consistent outcomes, it is easy to find other lines of evidence that might lead to a different hypothesis: that memory for *prediction-inconsistent* outcomes should be better compared to prediction-consistent outcomes. In the social literature, many investigations (that do not involve predictions) show that memory is often better for information inconsistent with what one already knows about targets [24, 31, 32]. For example, in one investigation participants learned about social targets who displayed specific characteristics (e.g., warmth, openness to experience) before then seeing additional information about targets that was either consistent or inconsistent with what they learned initially. When asked to recall information about targets, participants showed better memory for inconsistent compared to consistent information [33]. These findings parallel other theoretical and empirical work suggesting that inconsistent information about social targets is typically better remembered than consistent information [34–37]. Indeed, comprehensive meta-analyses have found that across a wide range of empirical works, memory for inconsistent information is generally better than information that is consistent with what one knows about targets [38, 39]. Given this past work, it may be surprising that Frankenstein et al. [29] did not find strong evidence for improved memory for prediction-inconsistent outcomes (although see below for one aspect of that data that hinted at a way to improve memory for prediction-inconsistent outcomes). In other domains, bountiful evidence suggests that when people (or even animal models) make predictions that are incorrect (i.e., prediction error), this can trigger deeper processing of the unexpected outcome [40–43]. Importantly, neurobiological evidence on prediction errors in animal models as well as in humans suggest that such errors exert their strongest effects on cognitive processes when the task is highly salient to the self [43, 44]. This is relevant because in Frankenstein, et al. [29] participants were making predictions about targets in a task that had low salience to the self (e.g., participants were making predictions about targets in a task that did not involve the self). Because the task in Frankenstein et al. [29] had low salience to the self, it is possible the task did not induce strong prediction errors in participants, which in turn may have reduced the likelihood of finding better memory for prediction-inconsistent outcomes, if such a memory advantage exists. Thus, in the current investigation, we worked to increase the self-salience of the prediction task to advance understanding of the relationship between prediction outcome and episodic memory. Specifically, participants were given a scenario that involved social targets as well as the self, and were asked to make a prediction of what behavior the targets would do in that scenario. We were most interested in the possibility that making predictions more relevant to the self would lead to improved memory for prediction-inconsistent outcomes.

Although Frankenstein et al. [29] showed clear evidence of enhanced memory for prediction-consistent outcomes, there was one aspect of the data that hinted at a way to improve memory for prediction-inconsistent outcomes. In the procedures of Frankenstein et al. [29], participants made expectancy judgments immediately after learning the behavior that social targets actually performed (i.e., prediction outcome). Specifically, after participants made their predictions and were shown the behaviors targets performed, participants then subjectively reported whether they expected that particular outcome (yes or no). Frankenstein included the expectancy rating to ensure that participants were attending to outcomes, especially the unexpected outcomes, which could make participants more likely to engage in further processing of the unexpected outcome (after they learned the prediction outcome). Aligned with this idea, results showed that memory was better for prediction-inconsistent outcomes when participants subjectively rated the outcome (i.e., what the target actually did) as unexpected relative to expected. Overall, the results of Frankenstein suggest that although memory for prediction-consistent outcomes is better in general, memory for prediction-inconsistent outcomes may be enhanced, but only when those outcomes are subjectively rated as unexpected by the participant. In this investigation, we examine memory for prediction-consistent and prediction-inconsistent outcomes, as a function of expectancy (whether prediction outcomes are expected or unexpected) to better understand how the outcome of predictions is subsequently remembered in a task that was designed to increase salience to the self compared to past work [29].

In this experiment, we examine memory for prediction-consistent versus prediction-inconsistent outcomes to better understand how the outcome of predictions are subsequently stored in episodic memory. To do so, participants first learned core traits about social targets. Then participants imagined a scenario involving the self and the social target before making a prediction about which of two behaviors the target would likely perform. Immediately after making each prediction, participants learned the behavior the target actually performed (i.e., prediction outcome) which was either consistent or inconsistent with predictions. Participants then made a subjective expectancy judgment on whether that outcome was expected or unexpected, before then completing a memory test for the prediction outcome. We have two hypotheses in this investigation. First, we hypothesized that participants would show better memory for prediction outcomes that are consistent (prediction-consistent outcomes) with prior knowledge compared to outcomes that are inconsistent, which is in line with past work on predictions [29] as well as other work in the social literature demonstrating consistent information about targets is well-remembered [14–18, 20, 21, 23–25, 45, 46]. However, because we made the prediction task more salient to the self than prior work [29], an alternative possibility is that prediction-inconsistent outcomes might be better remembered than prediction-consistent outcomes. Such a finding would be aligned with work showing prediction errors (such as the prediction-inconsistent outcomes) can induce deeper processing of unpredicted events [40–42], especially when salience to the self is higher [43, 44]. Finding better memory for prediction-inconsistent outcomes would be in line with other bodies of work showing that inconsistent information about targets sometimes shows an advantage in memory [33, 35, 39]. Second, because Frankenstein et al. [29] found better memory for prediction-inconsistent outcomes that were subjectively rated as unexpected compared to expected, we further hypothesized to find better memory for prediction-inconsistent outcomes when such outcomes were subjectively endorsed as unexpected, relative to expected, under task conditions where the salience to the self was higher than in past work. Such a finding would suggest a condition under which memory may be enhanced for prediction-inconsistent outcomes. Overall, the results of this investigation will advance understanding of the relationship between future thinking, specifically making predictions, and episodic memory. Further, the results of this investigation will add to literature on future thinking by showing how the outcomes of predictions are remembered after predicted events play out, which has not been the focus of much work in the past.

## Methods

### Participants

Thirty-two participants (16 females; age: *M* = 19.00, *SD* = 1.87*)* were recruited from the University of Illinois at Chicago subject pool. Power analyses using results from piloting showed that a sample of 17 participants would be sufficient to achieve a power of 0.80 at an alpha of 0.05.^1^ Participants gave their informed consent and were given course credit for participating in the experiment, in accord with relevant guidelines and regulations approved by the Institutional Review Board at the University of Illinois at Chicago.

### Stimuli

Stimuli consisted of 40 face-name pairs (half female, half male), 400 behavioral sentences (adapted from [47]), and 80 scenarios. Photos of neutral faces were taken from the Chicago Faces Database [48]. The behavioral sentences implied one of two traits: warmth or openness to experience. For example, “This person helps elderly neighbors with housework” is a behavioral sentence implying warmth. For each trait, half of the behaviors were high on that trait, whereas the other half were low on that trait (e.g., high warmth, low warmth, etc.). The 80 scenarios used in this study provided a context in which participants could make predictions about targets (e.g., “Imagine you are at an art museum with this person.”).

### Procedure

The experiment took place in a single laboratory session. In this investigation there were three experimental phases: learning, prediction and outcome, and recognition (memory phase). The phases were completed on a computer using E-prime software (Psychology Software Tools, Pittsburgh, USA). Stimuli were presented on a computer monitor with sentences in white font on a black background. Participants received instructions at the beginning of each phase and were given an opportunity to ask questions if they were unsure about task instructions.

Participants first completed the learning phase. The purpose of the person learning phase was for participants to learn the core trait (high warmth, etc.) associated with each target. In this phase, participants were shown 40 face-name pairs of social targets presented one at a time. In each learning phase trial, participants saw the target’s name, picture (i.e., face), and six behavioral sentences associated with the target. The behaviors for each social target implied one trait: either (high or low) warmth or openness to experience. Targets and their respective behavioral sentences were presented twice during learning to ensure that participants were learning the core trait associated with each social target, as we have done before [29]. The 40 targets were presented once, and then were presented a second time in a different random order. Immediately after the person learning phase, participants completed a pen-and-paper manipulation check that assessed how well participants learned the trait-implying sentences associated with each target. The manipulation check consisted of seven behavioral sentences per target. Some of the sentences were originally associated with the target and others were not. The behaviors that were not previously associated with the target were taken from the opposite dimension of the same trait. For example, if participants learned a target was high in warmth (i.e., kind and considerate), the “incorrect” behaviors implied low warmth (i.e., unkind or neglectful). Participants chose “TRUE” for behaviors that were consistent with the previously learned information and “FALSE” for behaviors not previously associated with the target.

**Fig. 1:**
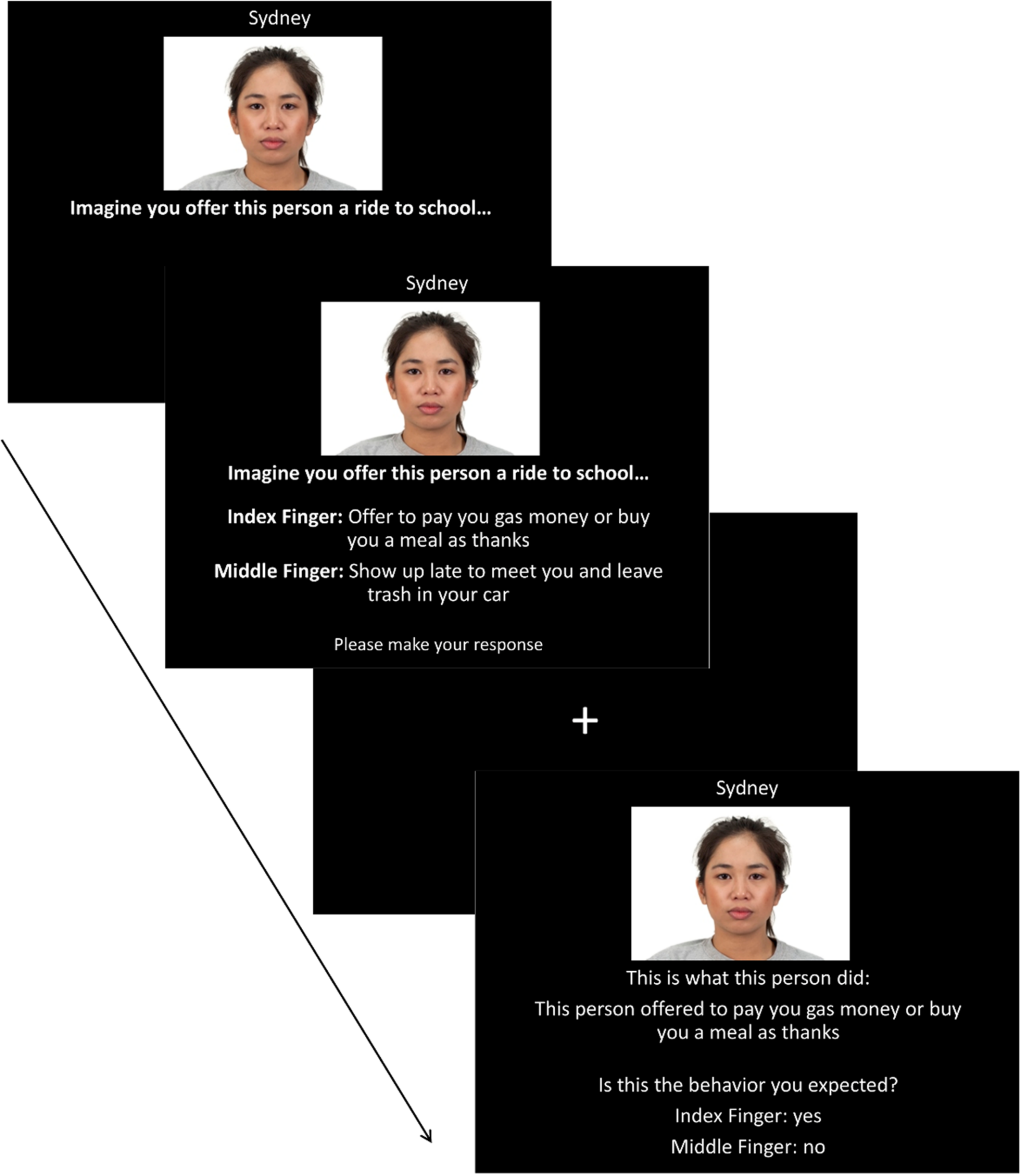
Prediction and outcome phase procedure. Participants were shown a scenario and two behaviors, and then made a prediction about which behavior the target would be more likely to do (based on previously learned information). After making the prediction, participants were shown the behavior the target actually performed (outcome) and indicated whether they expected the target to do this behavior (expectancy)

Next, participants completed the prediction and outcome phase (see Fig. 1). In this phase, participants predicted the future behavior of targets, found out what the target actually did (i.e., prediction outcome), and then made a subjective expectancy decision on whether they expected that outcome (yes or no). Overall, participants completed 80 trials in this phase of the experiment (two separate predictions for each social target). In each prediction and outcome phase trial, participants were shown a social target and asked to imagine a particular scenario involving themselves and the target (e.g., “Imagine you are at an art museum with this person”). After pressing the space bar to continue (self-paced), two behavioral sentences appeared below the scenario depicting two possible actions the social target might perform in that scenario. One of the behaviors was consistent with previously learned information (e.g., high warmth, etc.) about the target, and the other was inconsistent (taken from the opposite dimension of the same trait, e.g., low warmth, etc.). Participants were instructed to use their prior knowledge of targets to predict which behavior the social target would most likely perform in the given scenario (participants pressed “v” on a keyboard to predict the first behavior or “b” to predict second behavior). We specifically asked participant to imagine the scenario to make it more likely participants were truly making a simulation/prediction about the target for each trial. Immediately after making the prediction, there was an intervening fixation cross (250 ms) and then participants were shown the prediction outcome, which was the behavior that the social target actually performed. Participants then indicated whether or not the outcome was what they expected (“v” for yes, “b” for no), which served as the subjective *expectancy* rating, as we have done before [29].^2^ Because half of the prediction outcomes (behaviors targets performed) were consistent and half inconsistent with the core trait associated with targets, we balanced the prediction outcomes so that for half the targets the consistent outcome was presented first, and for the other half of targets the inconsistent outcome was presented first.

After the prediction and outcome phase, participants completed the recognition phase (memory test). Participants completed 80 recognition trials (one for each trial in the prediction and outcome phase). On each trial, participants saw the same scenarios, social target, and two behaviors that were presented in the prediction and outcome phase.^3^ Participants were asked to recognize the outcome of the prediction, that is, the actual behavior the social target performed. Instructions explicitly stated to participants that they were not to make another prediction, but rather they were choosing the behavior that the social target actually performed (i.e., what the target actually did).

## Results

In this section, we report data from the learning phase (i.e., manipulation check), the prediction and outcome phase as well as the recognition phase (i.e., memory) of this experiment. In the learning phase, participants accurately remembered the behaviors associated with targets 82% (SD = 0.03) of the time as assessed by the manipulation check, suggesting that participants were learning the behaviors associated with targets. In the prediction and outcome phase, participants predicted the outcome consistent with the core trait of targets 67% of the time (*SD* = 0.12), which suggests participants were using the information they learned about each target to make predictions.

**Fig. 2:**
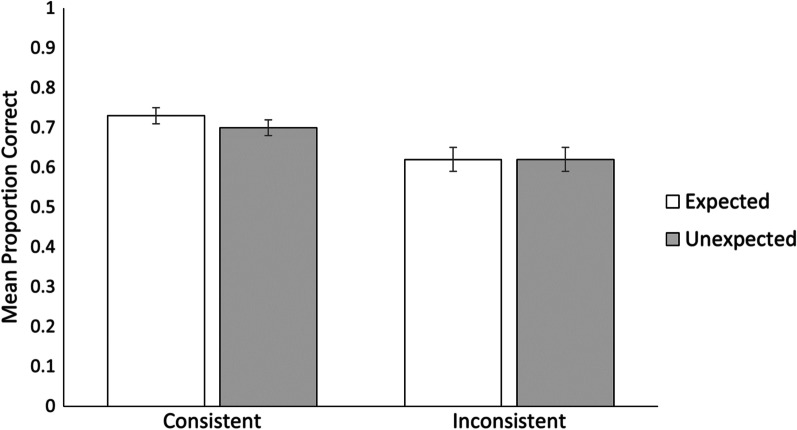
Mean proportions of recognition accuracy by consistency and expectancy. Error bars represent standard error of the mean. There was a main effect of consistency, with the likelihood of remembering consistent outcomes being higher than inconsistent outcomes. There was no main effect of expectancy and no interaction between the two variables

Turning to the memory data, we performed mixed-effects logistic regressions using the *GAMLj* module in *jamovi* to examine mean accuracy as a function of consistency and expectancy. To do this, we coded the memory data for accuracy on a trial-by-trial basis (0—incorrect, 1—correct). A trial was accurate if the participant correctly remembered the action the social target actually performed (i.e., prediction outcome). In all models, Subjects and Items were included as random effects, while Consistency (consistent versus inconsistent) and Expectancy (expected versus unexpected) were included as fixed effects. Subjects were included as a random effect to account for potential individual differences between participants, such as differences in memory capability. Items were included as a random effect to ensure that any individual stimuli were not driving effects rather than our primary experimental manipulation. We report two regression models. In the first logistic regression, we analyze how memory is affected by the consistency manipulation and participants’ expectancy ratings. For the second regression, we ran the same analysis but we only included trials where participants’ predictions reflected accurate learning of social targets (i.e., where participants predicted the outcome that was consistent with the core trait of the target). Turning to the first regression model, Fig. 2 shows the mean odds of recognition accuracy as a function of consistency and expectancy. We found a main effect for consistency, *χ*^2^(1) = 19.06, *p* < 0.001, with participants more likely to remember consistent outcomes (*M*_prob_ = 0.72, *SE* = 0.02) than inconsistent outcomes (*M*_prob_ = 0.62, *SE* = 0.02), aligned with our first hypothesis that memory would be better for prediction-consistent relative to prediction-inconsistent outcomes. There was no main effect for expectancy, *χ*^2^(1) = 0.46, *p* = 0.50, which shows that expected and unexpected outcomes were remembered with equal likelihood (*M*_prob_ = 0.68, and *M*_prob_ = 0.66, respectively). Importantly, there was no interaction between consistency and expectancy, *χ*^2^(1) = 1.00, *p* = 0.32. See Table 1 for the full statistical model.

**Table 1:** Statistical information for fixed and random effects of mixed-effects logistic regression

Fixed effects parameter estimates								
					95% Exp(B) confidence interval			
Names	Effect	Estimate	SE	Exp(B)	Lower	Upper	z	*p*
(Intercept)	(Intercept)	0.70	0.08	2.02	1.71	2.38	8.45	< .001
Consistency	Inconsistent–consistent	− 0.43	0.09	0.65	0.54	0.79	− 4.37	< .001
Expectancy	Unexpected–expected	− 0.06	0.09	0.94	0.79	1.12	− 0.68	0.50
Consistency * expectancy	Inconsistent–consistent * unexpected-expected	0.17	0.17	1.19	0.85	1.68	1.00	0.32

Random components			
Groups	Name	SD	Variance
Item	(Intercept)	0.30	0.09
Subject	(Intercept)	0.38	0.14
Residuals		1.00	1.00

**Fig. 3:**
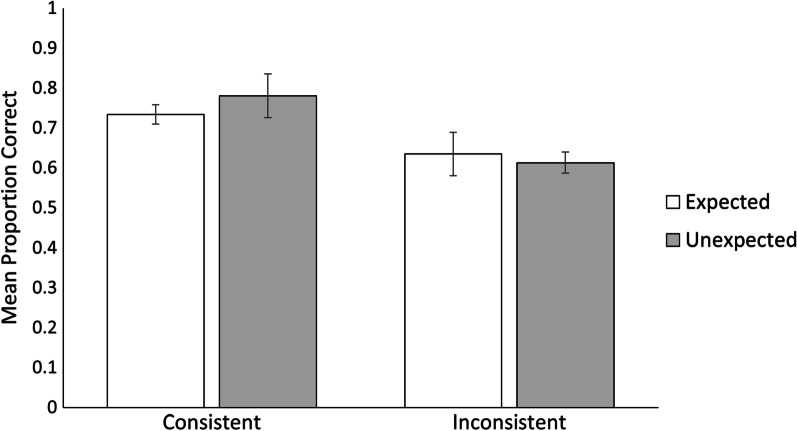
Mean proportions of recognition accuracy by consistency and expectancy for correct prediction trials. Error bars represent standard error of the mean. There was a main effect of consistency, with the likelihood of remembering consistent outcomes being higher than inconsistent outcomes. There was no main effect of expectancy and no interaction between the two variables

Because participants’ predictions did not fully match our a priori consistency manipulation (i.e., participants predicted the outcome that was consistent with the core trait of the target 67% of the time), we ran a second model using only trials in which participants chose the outcome that was aligned with the core trait of targets. Figure 3 shows the mean probabilities for this analysis. Results revealed a significant main effect of consistency, *χ*^2^(1) = 9.84*, p* = 0.002, where participants were more likely to remember prediction-consistent outcomes (*M*_prob_ = 0.76, *SE* = 0.03) than prediction-inconsistent outcomes (*M*_prob_ = 0.62, *SE* = 0.03). There was no main effect of expectancy, *χ*^2^(1) = 0.17*, p* = 0.68, and no interaction, *χ*^2^(1) = 0.71*, p* = 0.40. Table 2 shows results for the full model.

**Table 2:** Statistical information for fixed and random effects of mixed-effects logistic regression (correct prediction trials only)

Fixed effects parameter estimates									
						95% Exp(B) confidence interval			
Names	Effect	Estimate	SE		Exp(B)	Lower	Upper	z	*p*
(Intercept)	(Intercept)	0.82	0.13	2.28		1.79	2.91	6.61	< .001
Consistency	Inconsistent–consistent	− 0.64	0.20	0.53		0.36	0.79	− 3.14	0.002
Expectancy	Unexpected–expected	0.08	0.20	1.08		0.73	1.60	0.41	0.68
Consistency * expectancy	Inconsistent–consistent * unexpected–expected	− 0.35	0.42	0.71		0.31	1.60	− 0.84	0.40

Random components			
Groups	Name	SD	Variance
Item	(Intercept)	0.25	0.06
Subject	(Intercept)	0.36	0.13
Residuals		1.00	1.00

## Discussion

In this investigation, we examined memory for prediction-consistent and prediction-inconsistent outcomes to better understand the relationship between predictions, a type of future thinking, and episodic memory. We did so in a prediction task where we increased salience to the self relative to prior work [29]. We have two primary findings in this investigation. First, we observed better memory for prediction-consistent compared to prediction-inconsistent outcomes, which is aligned with schema accounts that suggest information that is consistent with existing memory stores is generally easier to integrate into memory than information that is inconsistent [26–28]. This extends past work demonstrating an intriguing relationship between predictions and episodic memory, where outcomes that are consistent with predictions (and the current contents of memory) are remembered better than inconsistent outcomes [29]. Second, we found no evidence that memory was better for prediction-inconsistent outcomes when those outcomes were subjectively rated as unexpected compared to expected, suggesting that even when inconsistent outcomes are surprising or unexpected, this is insufficient to improve memory for those outcomes. Overall, findings of this investigation extend understanding on the relationship between future thinking and memory, by examining how outcomes that are consistent or inconsistent with what one predicts are subsequently represented in episodic memory.

Predictions interest researchers because of their implications for learning, planning, and many other cognitive processes [2]. People frequently make predictions about the world [11], and at times those predictions are accurate (or consistent with what occurs), whereas other times they are inaccurate (or inconsistent with what occurs). In this investigation, our primary finding was that subsequent episodic memory was better for prediction-consistent relative to prediction-inconsistent outcomes after the predicted event occurred. This finding is in line with past work showing that consistent information about social targets is better remembered than inconsistent information [17–19, 21–25], and further in line with schema accounts that posit that consistent information is easier to integrate into memory than inconsistent information [26–28, 50]. Finding better memory for prediction-consistent outcomes implies that participants were using their existing knowledge to process new information about targets. Interestingly, past work in the memory domain suggests many benefits to using existing knowledge to process new information. For instance, theoretical work on so-called “resource” accounts in memory suggest that using prior knowledge to process new information is cognitively efficient [51–53]. Specifically, such resource accounts suggest processing information that is related or familiar to what one knows (such as consistent information) is less cognitively and attentionally demanding than processing unrelated or unfamiliar information, which importantly means there are additional cognitive resources available for further flexible use. Thus, our finding of a prediction-consistent advantage in memory may reflect an adaptive function that operates to reduce cognitive demands when thinking about social targets, which importantly may leave additional resources available to process other complex information associated with social interactions.

The fact that we found better memory for prediction-consistent outcomes and no evidence for enhanced memory for prediction-inconsistent outcomes, even outcomes subjectively rated as unexpected, implies that people may be slow to change or update existing schematic representations about targets. In the social domain, other lines of work suggest that people can be slow to update their representations about targets, such as work showing that impressions formed of others tend to be slow to change once formed [54]. Overall, it may be our finding of improved memory for prediction-consistent outcomes, may reflect an adaptive process in memory, at least for social targets, where schemas for targets are strongly relied on to process new information, and thus, such schematic representations are resistant to change. This is in line with work showing that schematic information has a strong effect on memory [55, 56]. Understanding adaptive processes in memory is important pursuit [57–62], and the results of this investigation add to that knowledge.

In this investigation, we found better memory for prediction-consistent outcomes in a task where salience to the self was higher than in past work [29]. Despite this enhanced self-salience, we observed no evidence for improved memory for prediction-inconsistent outcomes, even those subjectively rated as unexpected or surprising. Given past work in the memory domain suggesting that increasing salience to the self has a strong effect on episodic memory (i.e., self-reference effects; [63–75]), and additional neurobiological evidence that errors produced in tasks with higher salience to the self can induce stronger prediction errors which can lead to additional processing of discrepant information [43, 44], we hypothesized that memory for prediction-inconsistent outcomes would be enhanced when those outcomes were endorsed as unexpected relative to expected, but that is not what we found. One possible reason we saw no hint of improved memory for prediction-inconsistent outcomes may be that the outcomes (e.g. behaviors) we used in this investigation were not sufficiently surprising to truly induce strong prediction errors that could, in turn, affect subsequent memory. In line with this idea, past meta-analytic work has shown that memory for inconsistent outcomes about targets is better when that inconsistent information is strongly, compared to weakly inconsistent with prior information. For example, Rojahn and Pettigrew [38] reported stronger inconsistency effects (better memory for inconsistent relative to consistent information) when stimuli were moderately inconsistent with prior information, whereas there were no inconsistency effects in experiments where stimuli were low in inconsistency with respect to prior information about targets. Turning back to the present study, the inconsistent outcomes we used (e.g., seeing a high openness to experience target engage in a low openness behavior) may not have been sufficiently surprising to truly induce deeper processing of the inconsistent information. Although speculative, it may take much stronger inconsistent, or surprising, information (such as seeing an “honest” target engage in cheating behaviors) before such inconsistent information is truly incorporated into memory.

Given bountiful empirical [31–37] and meta-analytic work [38, 39] showing that inconsistent information about social targets often shows a memory advantage over consistent information, and additional evidence that errors in prediction (e.g., prediction errors) can induce deeper processing of information [40, 42], it is somewhat surprising that we found no evidence for enhanced memory for prediction-inconsistent outcomes, even those subjectively rated as unexpected. After all, prediction errors leading to improved memory for prediction-inconsistent information may be useful, because remembering such outcomes might allow for memory to be updated so that future predictions about targets might be more accurate. Interestingly, neurobiological evidence examining prediction errors suggests that there are different types of prediction errors related to processing different types of stimuli. Importantly, some work suggests that some of the strongest prediction errors are related to the presence (or unexpected absence) of rewards [43]. This is relevant to the current study because we did not include rewards (e.g., money, etc.) as part of our experimental procedures. Thus, it may be that we did not observe evidence for enhanced memory for prediction-inconsistent outcomes because our procedures did not induce prediction error that were tied to rewarding stimuli. Specifically, without rewards it is possible that participants were not sufficiently motivated to further process discrepant information (i.e., prediction-inconsistent outcomes). Future work might extend the present findings by including more rewarding stimuli to investigate conditions under which memory for prediction-inconsistent outcomes may be enhanced.

In this investigation, we found clear evidence that prediction-consistent outcomes were remembered better than prediction-inconsistent outcomes, which extends knowledge on the relationship between future thinking and memory. Recent theoretical advances on future thinking suggest that there are different types, or taxonomies, of future thinking. Specifically, this work suggests there are four broad types of future thoughts: predictions, simulations, planning, and intentions [76]. This taxonomic account further suggests that these different types of future thinking are constructed using the contents of episodic memory (memory for specific past episodes), semantic memory (less detailed and more abstracted memory representations), or both. This is relevant because in the current investigation, participants simulated a situation (e.g., “imagine you are at a museum with this person”) and then made predictions about behaviors using specific information (i.e., episodic memory) they learned about the targets in the person learning phase of the experiment. Thus, the results of this investigation represent an advance in knowledge about how future thoughts (specifically predictions and simulations) that are constructed from the contents of episodic memory representations are subsequently remembered. To further advance understanding of the relationship between future thinking and memory, future investigations might examine how outcomes related to other types of future thinking, such as intentions, are subsequently stored in memory. In addition, future work might also investigate how future thinking that is constructed from more semantic memory representations might be subsequently stored in memory to better understand the relationship between future thinking and memory. Understanding processes in episodic memory is an important scientific endeavor [59, 77–92] and the findings of this investigation add to that effort.

Overall, findings of this investigation advance knowledge about future thinking. Although much of the available literature on future thinking investigates *processes* involved in making predictions/simulations [2], much less work has investigated the extent that the outcomes of those predictions affect how events are remembered subsequently. Given that past work shows that memory for past experiences is central in future thinking (e.g., memory for past events is used to make predictions about the future), our findings help to advance knowledge in the domain of future thinking by showing how the outcome of predictions are remembered after predicted events occur (which in turn would affect future simulations). Further, although we used stimuli that were social in nature (e.g., social targets), we think the memory effects we observed (i.e., enhanced memory for prediction-consistent relative prediction-inconsistent outcomes) would generalize to non-social stimuli as well. Thus, future work might use similar procedures, but use non-social materials to further understand the relationship between future thinking and episodic memory.

Although the results of the current investigation suggest that memory is better for prediction-consistent compared to prediction-inconsistent outcomes, there are three limitations of the current investigation that are work describing. First, as part of our procedures we used the exact same behaviors in both the prediction and outcome phase as well as the recognition (memory) phase of this experiment. It is possible that in both experimental phases (prediction and outcome; recognition), participants were not following task instructions (i.e., making predictions about targets in the prediction and outcome phase; searching through memory and retrieving the behavior targets actually performed), and instead were simply selecting the behavior that was consistent with the core trait associated with targets from the learning phase of the experiment which could have affected our memory results. Although possible, we see this as less likely given that we trained participants extensively on task instructions for all phases of the experiment. Second, in this work, we showed enhanced memory for prediction-consistent outcomes in a task that induced higher self-salience relative to past work (Frankenstein et al., 2020); however, we did not include a low self-salience condition in our experimental design. To further understand the influence of self-salience on memory for prediction outcomes, future work should include both higher- and lower-self-salience conditions in the prediction task within the same experiment. Third, as part of our procedures we measured memory immediately after the prediction and outcome phase of the experiment. Given that some work suggests that memory effects for schematic information as well as memory effects for unexpected/surprising information differ after a period of consolidation [39, 93], it is possible that the memory effects we observed may have been different if we measured memory after a longer delay. In line with this idea, future work on this topic should include a longer delay period to better understand memory effects for prediction-consistent and prediction-inconsistent outcomes.

In conclusion, this investigation found that prediction-consistent outcomes were better remembered than prediction-inconsistent outcomes, implying that information consistent with what one already knows (e.g., schemas) shows an advantage in memory, which may reflect an adaptive process in memory for social information. Thus, our data offer further insight into how people make predictions about social targets and incorporate the outcomes of those predictions into memory. Overall, this work extends knowledge on the relationship between future thinking and episodic memory.

## Data Availability

Data are available from the corresponding author upon reasonable request.

## Footnotes

1 We performed this pilot in an independent sample of participants. Pilot results for our primary comparison of interest (memory for prediction-consistent versus prediction-inconsistent outcomes) showed an effect size of *d* = .92, but we used a more conservative effect size (*d* = .6) for our power analysis.

2 In addition to following the procedures used in Frankenstein, et al. (2020), we chose to include the subjective expectancy rating, because we wanted to make it more likely that participants were attending to whether the prediction outcome was consistent or inconsistent with the core trait associated with each target. Thus, by including the expectancy measure, we were creating a check to make it more likely participants were noting potential discrepancies between the prediction outcome (behavior the target actually performed) and the core trait associated with each target. Further, some work suggests that making retrospective judgments about stimuli (e.g., making decisions about materials after exposure to stimuli) can induce post-event processing that can lead to enhanced memory for unexpected or surprising information (in line with work on hindsight bias; Ash, 2009; Schutzwohl, 1998). Therefore, by including our subjective expectancy measure, we increased our likelihood of finding improved memory for inconsistent and unexpected events in this investigation.

3 When designing this experiment, we chose to include the same behaviors in both prediction and outcome phase as well as for the recognition phase for a specific memory-related reason. In the memory literature there is ample evidence to suggest that when making recognition decisions, participants can “recollect” a past experience (i.e., remember specific details associated with past events), or they can rely on familiarity (i.e., the feeling that they’ve encountered something before without actually retrieving specific details) in service of making recognition judgments [49]. This is relevant because if we had shown a truly novel behavior in the recognition phase of the experiment, participants could have made memory judgments based purely on familiarity (choosing the behavior that felt “familiar” because they had seen it before) instead of actually retrieving which behavior the target performed. Because the experimental approach we used showed the same behaviors in both the prediction and outcome phase as well as the recognition phase, this means that both behaviors presented in the recognition phase of the experiment would be associated with familiarity (since both behaviors had been seen before). Thus, our procedures make it less likely that participants could simply have been making recognition judgments based on familiarity without actually remembering prediction outcomes.
